# Network pharmacology and bioinformatics analysis identifies potential therapeutic targets of *Naringenin* against COVID-19/LUSC

**DOI:** 10.3389/fendo.2023.1187882

**Published:** 2023-06-06

**Authors:** Wen-yu Wu, Xin Jiao, Wen-xin Song, Peng Wu, Pei-qi Xiao, Xiu-fang Huang, Kai Wang, Shao-feng Zhan

**Affiliations:** ^1^ The First Clinical Medical College of Guangzhou University of Chinese Medicine, Guangzhou, China; ^2^ The First Affiliated Hospital of Guangzhou University of Chinese Medicine, Guangzhou, China; ^3^ Lingnan Medical Research Center of Guangzhou University of Chinese Medicine, Guangzhou, China

**Keywords:** COVID-19, lung squamous cell carcinoma, *naringenin*, network pharmacology, bioinformatics

## Abstract

**Background:**

Coronavirus disease 2019 (COVID‐19) is a highly contagious respiratory disease that has posed a serious threat to people’s daily lives and caused an unprecedented challenge to public health and people’s health worldwide. Lung squamous cell carcinoma (LUSC) is a common type of lung malignancy with a highly aggressive nature and poor prognosis. Patients with LUSC could be at risk for COVID-19, We conducted this study to examine the potential for naringenin to develop into an ideal medicine and investigate the underlying action mechanisms of naringenin in COVID-19 and LUSC due to the anti-viral, anti-tumor, and anti-inflammatory activities of naringenin.

**Methods:**

LUSC related genes were obtained from TCGA, PharmGKB, TTD,GeneCards and NCBI, and then the transcriptome data for COVID-19 was downloaded from GEO, DisGeNET, CTD, DrugBank, PubChem, TTD, NCBI Gene, OMIM. The drug targets of *Naringenin* were revealed through CTD, BATMAN, TCMIP, SymMap, Chemical Association Networks, SwissTargetPrediction, PharmMapper, ECTM, and DGIdb. The genes related to susceptibility to COVID-19 in LUSC patients were obtained through differential analysis. The interaction of COVID-19/LUSC related genes was evaluated and demonstrated using STRING to develop a a COX risk regression model to screen and evaluate the association of genes with clinical characteristics. To investigate the related functional and pathway analysis of the common targets of COVID-19/LUSC and Naringenin, KEGG and GO enrichment analysis were employed to perform the functional analysis of the target genes. Finally, The Hub Gene was screened and visualized using Cytoscape, and molecular docking between the drug and the target was performed using Autodock.

**Results:**

We discovered numerous COVID-19/LUSC target genes and examined their prognostic value in LUSC patients utilizing a variety of bioinformatics and network pharmacology methods. Furthermore, a risk score model with strong predictive performance was developed based on these target genes to assess the prognosis of LUSC patients with COVID-19. We intersected the therapeutic target genes of naringenin with the LUSC, COVID-19-related targets, and identified 354 common targets, which could be used as potential target genes for naringenin to treat COVID-19/LUSC. The treatment of COVID-19/LUSC with naringenin may involve oxidative stress, anti-inflammatory, antiviral, antiviral, apoptosis, immunological, and multiple pathways containing PI3K-Akt, HIF-1, and VEGF, according to the results of the GO and KEGG enrichment analysis of these 354 common targets. By constructing a PPI network, we ascertained AKT1, TP53, SRC, MAPK1, MAPK3, and HSP90AA1 as possible hub targets of naringenin for the treatment of COVID-19/LUSC. Last but not least, molecular docking investigations showed that naringenin has strong binding activity in COVID-19/LUSC.

**Conclusion:**

We revealed for the first time the pharmacological targets and potential molecular processes of naringenin for the treatment of COVID-19/LUSC. However, these results need to be confirmed by additional research and validation in real LUSC patients with COVID-19.

## 1 Introduction

An acute respiratory infectious illness called COVID-19 is brought on by the SARS-CoV-2 virus (1). Coughing, a sore throat, fever, arthralgias, myalgias, exhaustion, and headache are among the usual COVID-19 symptoms. Acute respiratory distress syndrome (2, 3), shock (4, 5), metabolic acidosis (6), and multiple organ failure (7, 8) may develop in patients with comorbidities. As of November 28, 2022, with 636,440,663 confirmed COVID-19 cases and 6,606,624 deaths reported globally (9). Despite the development of many COVID-19 vaccines and the initiation of mass vaccinations, the number of infections is still continuously increasing (10). Additionally, several antiviral medications have been used to treat COVID-19, such as remdesivir, but they have not been generally adopted because of their high cost and requirement for intravenous administration (11). Studies have demonstrated that cancer patients, including those with lung cancer (12), esophageal cancer (13), colorectal cancer (14), and breast cancer (15), among others, are more susceptible to SARS-CoV-2 infection and have a higher fatality rate (16, 17). Therefore, it is crucial to screen beneficial, affordable, and widely accessible drugs against COVID-19. Lung cancer is one of the most common malignant tumors in humans and has the highest mortality rate worldwide, with 1.6 million fatalities per year (18, 19). The hospital served as the primary infection source during the early stages of the outbreak, and patients with lung cancer who were admitted there for antitumor therapy significantly increased their risk of COVID-19 (20). The majority of patients with lung cancer are immunosuppressed (21), and there is a pressing need for effective drugs to treat lung cancer and COVID-19 with few side effects.

**Figure 1:**
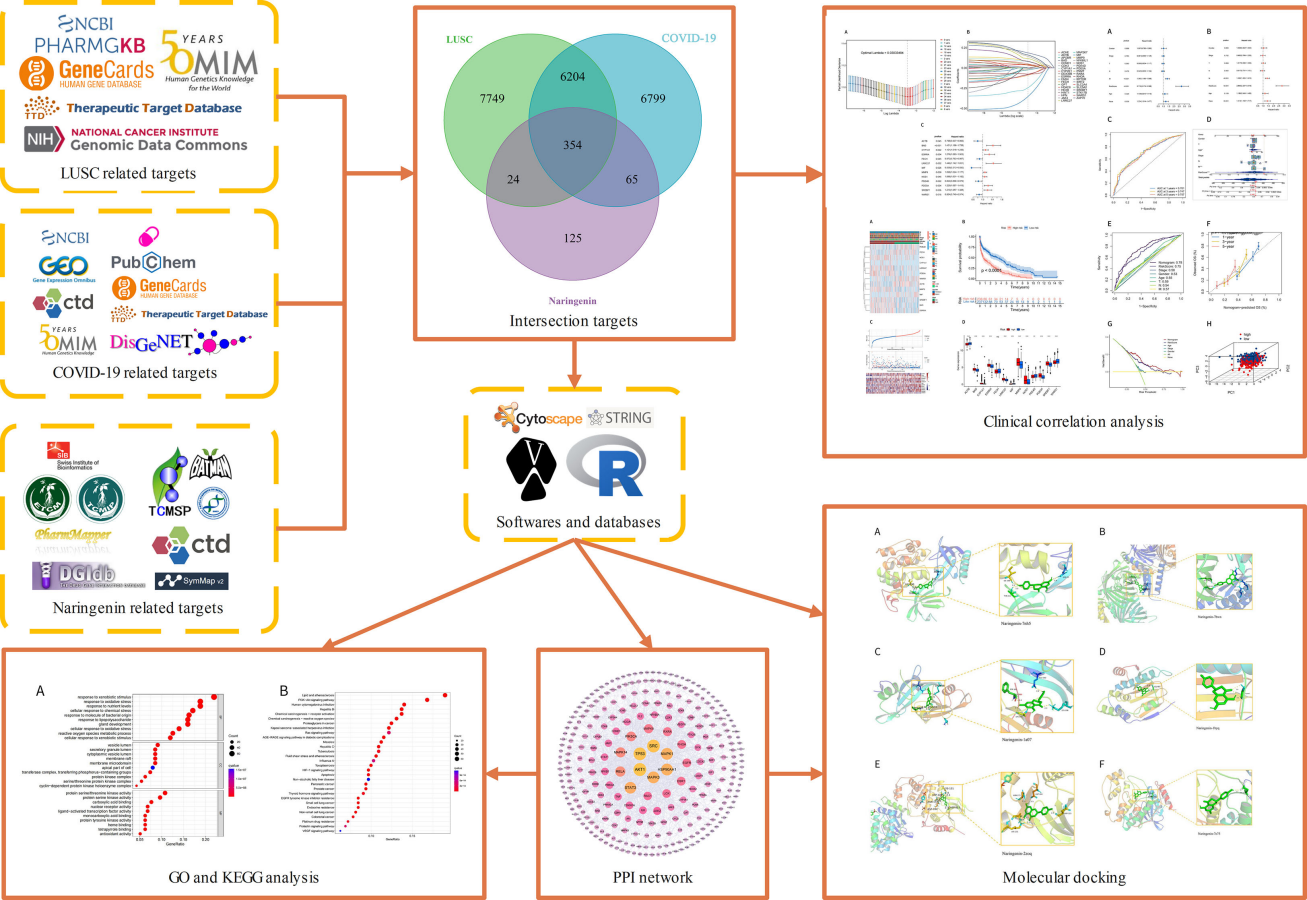
Flow of the entire investigation.

*Naringenin* is a common dietary flavanone found in citrus fruits such as oranges, bergamots, lemons, and grapefrui (22). *Naringenin* has a molecular formula C15H12O5 and is chemically named 2,3-dihydro-5,7-dihydroxy-2-(4-hydroxyphenyl)4H-1-benzopyran-4-one (23). Pharmacologically, it has anticancer, antimutagenic, antioxidant, antiproliferative, and antiatherogenic activities (24). *Naringenin* is Commonly used for the treatments of diabetic (25), cognition deficits (26), bronchial pneumonia (27), nonalcoholic steatohepatitis (28, 29), and neurodegenerative diseases (30–32). Naringin possesses antiviral and anti-inflammatory properties, which include lowering viral replication and cytokine production, according to recent studies (33). By entering human cells through the angiotensin-converting enzyme 2(ACE2) receptor and Transmembrane Serine Protease 2(TMPRSS2) (34), SARS-CoV-2 can cause infection. When a virus infects a host, it causes the host to produce and release more inflammatory cytokines, which can boost immune activity and cause tissue damage (35). A growing body of research suggests that antiviral therapy may help treat COVID-19 symptoms as well as those that reduce inflammatory responses (36, 37). Through both transcriptional and post-transcriptional processes, *naringenin* can reduce the generation of inflammatory molecules (38). Macrophages have a crucial role in the pathogenesis of COVID-19 because they can detect infections, react to them, and create inflammatory cytokines and chemokines (39). Without affecting the toll-like receptor(TLR) cascade, *naringenin* decreased the generation of TNF and IL-6 by macrophages and T cells in animal experimental models (39). A recent study suggests that *Naringenin* can function as having the potential inhibitor of SARS‐CoV‐2 main protease, *naringenin* may be considered as potential for preventing CoV replication (40, 41). In addition, *Naringenin* exhibits the role of treatment of lung cancer by reducing tumor cell proliferation, migration, and invasion while increasing apoptosis (42, 43). As far as we can tell, naringin’s molecular mechanisms and targeting have not been investigated in the treatment of COVID-19 in patients with LUSC. In this study, we examine the prognostic value of COVID-19-related genes in LUSC patients and further explore the potential anti-COVID-19/LUSC mechanisms of *naringenin* using network pharmacology and bioinformatics methods. Our findings offer some fresh perspectives on how *naringenin* works to treat COVID-19/LUSC. A clear graphical summary that detailed the entire study process was used in **Figure 1**.

## 2 Materials and methods

### 2.1 Ethics statement

Because the data were sourced from free databases, ethics committee approval was not necessary for this study.

### 2.2 *Naringenin* database building

PubChem (https://pubchem.ncbi.nlm.nih.gov/) comprises a wide range of chemical information from 750 data sources (44). The 2D structure, 3D structure, InChI, and canonical SMILES profiles of naringin were obtained from PubChem (44).

### 2.3 Identification of COVID-19/LUSC-associated genes

The transcriptome profiles of LUSC patients were downloaded from The Cancer Genome Atlas (TCGA) database (https://portal.gdc.cancer.gov/) on 19 November 2022, and the differentially expressed genes (DEGs) were screened and obtained using the ‘limma’ package of R-language(version 4.2.2)Bioconductor with P-value< 0.05 and |log2FC| >1 (45). DEGs were represented using volcano plots created with the R-language packages “ggpubr” and “ggthemes”. PharmGKB (https://www.pharmgkb.org), NCBI Gene (https://www.ncbi.nlm.nih.gov/) (46), Therapeutic Target Database (TTD, http://db.idrblab.net/), GeneCards (https://www.genecards.org/), and Online Mendelian Inheritance in Man (OMIM, https://www.omim.org/) (47) were also used to collect LUSC related targets.

The COVID-19-related targets were identified by examining the transcriptome RNA-seq data of COVID-19 (GSE171110 and GSE179850) from the Gene Expression Omnibus database (GEO, https://www.ncbi.nlm.nih.gov/geo) (48). After normalization and removal of the batch effect by using the “sva” package, the difference analysis was conducted with the criteria of adjusted P-value< 0.05 and |log2FC| > 1 by using the “limma” package (45). Furthermore, targets associated with COVID-19 were obtained by searching the following eight databases: 1) DisGeNET(http://www.disgenet.org/), 2) Comparative Toxicogenomics Database(CTD, http://ctdbase.org/), 3) DrugBank(https://go.drugbank.com/), 4) PubChem(https://pubchem.ncbi.nlm.nih.gov/), 5)Therapeutic Target Database, 6) GeneCards, 7) NCBI Gene, 8)Online Mendelian Inheritance in Man. Targets gathered from public databases and GEO datasets were combined. The targets of LUSC and COVID-19 were then intersected to create a gene set that is connected to COVID-19/LUSC.

### 2.4 Fishing of *naringenin*-related targets

From the following databases, many pharmacological targets connected to *naringenin* were gathered: 1) CTD (http://ctdbase.org/) (49), 2) Bioinformatics Analysis Tool for Molecular mechANism of Traditional Chinese Medicine(BATMAN, http://bionet.ncpsb.org.cn/batman-tcm/) (50), 3) Integrative Pharmacology-based Research Platform of Traditional Chinese Medicine (TCMIP, http://www.tcmip.cn/TCMIP/) (51), 4) Symptom Mapping (SymMap, https://www.Symmap.org/) (52), 5) Swiss Target Prediction (http://www.swisstargetprediction.ch/) (53), 6) Chemical Association Networks (STITCH, http://stitch.embl.de/) (53), 7) PharmMapper (http://www.lilabecust.cn/pharmmapper/) (54), 8) Encyclopedia of Traditional Chinese Medicine (ECTM, http://www.tcmip.cn/ETCM/) (51), and 9) Drug Gene Interaction Database (DGIdb, https://www.dgidb.org/) (55). The target genes were transformed to standard gene symbols by using the UniProt database (https://www.uniprot.org/) with the limitation of “Human species”. After deleting the duplicate data, there were 568 targets of *naringenin*.

### 2.5 Acquisition of *naringenin* targets in COVID-19/LUSC

Using the Venn diagram tool (http://bioinformatics.psb.ugent.be/webtools/Venn/) and Microsoft Excel, it was possible to further achieve the goal of removing the repeated targets among *naringenin*, LUSC, and COVID-19. Then, the intersection of *naringenin*-related targets and COVID-19/LUSC-related targets was then screened for common targets.

### 2.6 Construction of LUSC/COVID-19-related prognostic signature for lung squamous cell carcinoma patients

Univariate Cox analysis was performed on 354 common targets, and Lasso Cox regression was performed on significantly expressed genes based on a 1000 ten-fold cross-validation to identify COVID-19-related genes. Optimal prognostic genes were identified based on multivariate Cox regression analysis (P< 0.05), and the best model parameters were used for signature construction, followed by the calculation of risk scores.


Risk score=Exp gene1 × β gene1 + Exp gene2× β gene2+…Exp gene n×β gene n

### 2.7 Analysis of prognosis signature

Patients were divided into high- and low-risk groups based on median values of a risk score to determine the prognosis of the signature. We used the survival package to calculate overall survival (OS) for patients with LUSC in different groups and performed univariate and multivariate independent prognostic analyses to evaluate the independent prognostic value of the risk prediction signature. The pheatmap package was used to plot patient survival status and gene expression heatmap based on the risk scores. The survival ROC package was used to calculate the 1-, 3-, and 5-year area under the receiver operating characteristic curve(ROC) curve (AUC) of signature in the LUSC patients.

### 2.8 Construction of nomogram and validation of clinical subgroups

Nomograms were constructed for age, gender, T stage, N stage, M stage, and risk score using the survival and rms packages. Calibration curves were plotted to show the difference between the predicted and actual outcomes of the nomogram. Decision curve analyses are used to verify the accuracy of the signature in predicting the survival of patients with LUSC.

### 2.9 Principal component analysis

PCA analysis was performed using limma and scatterplot3d packages to explore the distribution of patients with the high and low-risk groups.

### 2.10 Analyses of the protein-protein interaction network and hub targets

PPI networks contribute to a better understanding of target-related pathogenesis at the protein level. Thus, the STRING 11.5b database (https://string-db.org/) was used to fabricate the PPI network and acquire hub targets. The organism was selected as “Homo sapiens” and the minimum required interaction score with a correlation degree ≥0.900 was the cut-off value (56). Subsequently, the PPI network was visualized and analyzed by Cytoscape 3.9.1 software (https://cytoscape.org/). The degree values in the PPI network were calculated by using the NetworkAnalyzer CytoNCA of Cytoscape 3.9.1 software. Then, targets with degree values higher than the median were filtered as hub targets (57).

### 2.11 Enrichment analyses for common targets

To investigate the related functional and pathway analysis of the common targets of COVID-19/LUSC and *Naringenin*, R packages such as “enrichplot”, “clusterProfiler” (58), “org.Hs.eg.db”, and “ggplot2” were used to perform GO functional analysis and KEGG pathway enrichment analysis (59).Biological processes (BP), cellular components (CC), and molecular functions(MF) were the three categories included in the GO analysis. For enrichment, q-value cutoff = 0.05 and p-value cutoff = 0.05 were set, and the output was utilized to construct the bubble chart (46, 60).

### 2.12 Molecular docking

The binding situation and interaction force of proteins and small molecules may be anticipated and acquired *via* molecular docking analysis. *Naringenin* was docked with the PPI network’s top six hub targets, which included RAC-alpha serine/threonine-protein kinase(AKT1), TP53-target gene 3 protein(TP53), Proto-oncogene tyrosine-protein kinase Src(SRC), Heat shock protein HSP 90-alpha(HSP90AA1), Mitogen-activated protein kinase 3(MAPK3), and Mitogen-activated protein kinase 1(MAPK1). *Naringenin*’s two-dimensional molecular structure was retrieved from the PubChem database (https://pubchem.ncbi.nlm.nih.gov/) (61), and its three-dimensional structure was created and optimized using the MM2 force field in ChemBioOffice software (version 2019) (62). Following that, *Naringenin*’s output ligand file was saved in mol2 format. The protein structures of the hub targets were obtained from the PDB database (https://www.rcsb.org/) (63). All water molecules and original ligands were removed from the structures using PyMOL software (https://pymol.org/2/) and saved as PDB files. AutoDockTools (Vina 1.5.6, http://autodock.scripps.edu/) was used to convert the ligand files and original protein receptor to PDBQT file format that be identified by the Autodock Vina software for further molecular docking. Lastly, PyMOL software was used to analyze and present all docking results (64).

## 3 Results

### 3.1 Targets identification of *naringenin* and COVID-19/LUSC

**Figure 2:**
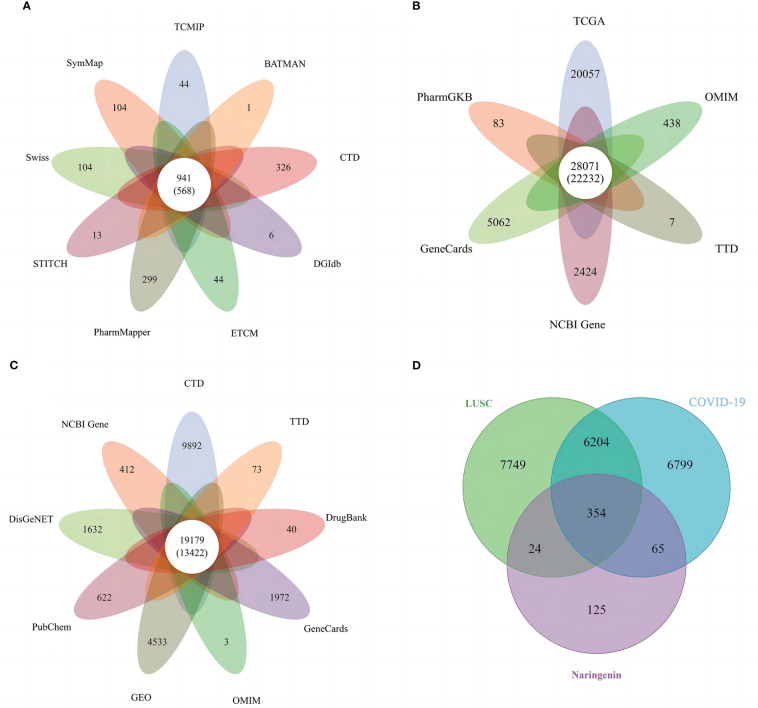
**(A)** Targets connected to naringenin in nine open-source databases. **(B)** Targets connected to LUSC in six open-source databases. **(C)** Targets connected to COVID-19 in nine open-source databases. **(D)** A Venn diagram demonstrating the common targets of naringenin, LUSC, and COVID-19.

**Figure 3:**
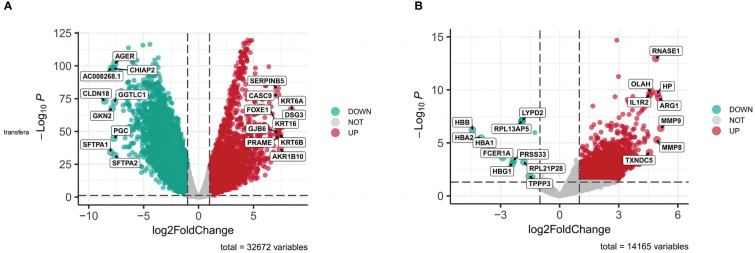
**(A)** DEGs volcano plots for LUSC. **(B)** DEGs volcano plots for COVID-19.

All targets of *naringenin* were obtained from Nine open-source databases, namely, BATMAN (1), CTD (326), DGIdb (6), ETCM (44), PharmMapper (299), STITCH (13), Swiss (65), SymMap (65), and TCMIP (44). And 568 targets related to *naringenin* were acquired after eliminating duplicate targets (**Figure 2A**). Subsequently, a total of 10649 DEGs (4042 upregulated and 6607 downregulated) of LUSC were identified from the TCGA. DEGs volcano plots for LUSC are displayed in **Figure 3A**. In addition, LUSC related genes collected from PharmGKB, NCBI Gene, TTD, GeneCards, and OMIM were 83, 2424, 7, 5062, and 438, respectively. By merging DEGs from the TCGA and LUSC-related genes from online platforms and removing duplication, we obtained 22232 LUSC related targets (**Figure 2B**). In total, 4533 DEGs (4561 upregulated and 17 downregulated) of COVID-19 were identified from the GEO. DEGs volcano plots for COVID-19 are displayed in **Figure 3B**. In addition, COVID-19 related genes retrieved from DisGeNET, CTD, DrugBank, PubChem, TTD, GeneCards, NCBI Gene, and OMIM were 1632, 9892, 40, 622, 73, 1972, 412, and 3, respectively. By merging DEGs from the GEO and LUSC-related genes from online platforms and removing duplication, we obtained 13422 COVID-19 related targets (**Figure 2C**). Finally, we acquired 354 common targets between *naringenin*, LUSC, and COVID-19, which were shown using the Venn diagram tool (**Figure 2D**).

### 3.2 Construction of LUSC/COVID-19-related prognostic signature

**Figure 4:**
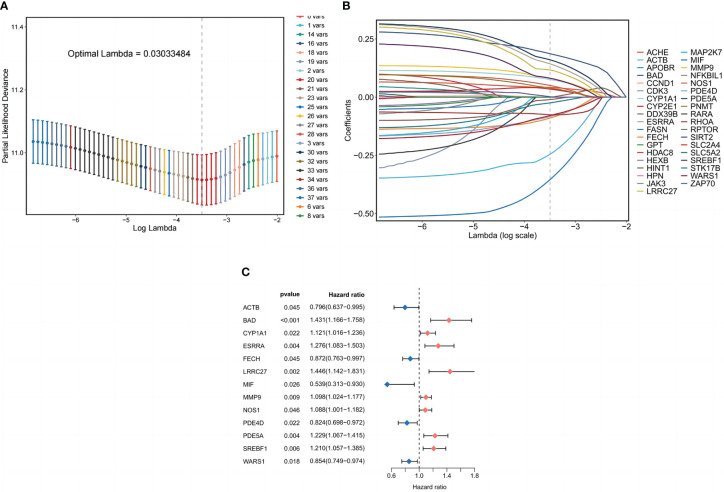
Prognostic model of LUSC patients. **(A)** Coefficient profiles plotted for LASSO regression analysis of LUSC. **(B)** Cross-validation error rate plotted for LASSO regression analysis of LUSC. **(C)** Partial presentation of prognostic related genes.

Cox regression analysis of 354 common target expression data and survival information was conducted using the coxph method in the “survival package” of the R language. The filtering standard was P<0.05, and 37 single-factor significant genes were obtained. Lasso regression analysis was performed on the above single-factor significant gene expression data, and Lasso regression plots and cross-validation plots were drawn (**Figures 4A, B**), and there were 20 Lasso regression significant genes based on the λ minimum value of LASSO Cox regression (0.03033484), Finally, 13 prognosis-related genes for constructing the model were identified using multifactorial Cox regression analysis, and forest plots were drawn (**Figure 4C**). Those involved in the model construction (BAD, CYP1A1, ESRRA, LRRC27, MMP9, NOS1, PDE5A, and SREBF1) with risk coefficients greater than 1 were defined as risk factors in LUSC, and their higher expression correlated with the worst OS of LUSC.

### 3.3 Analysis of prognosis signature

**Figure 5:**
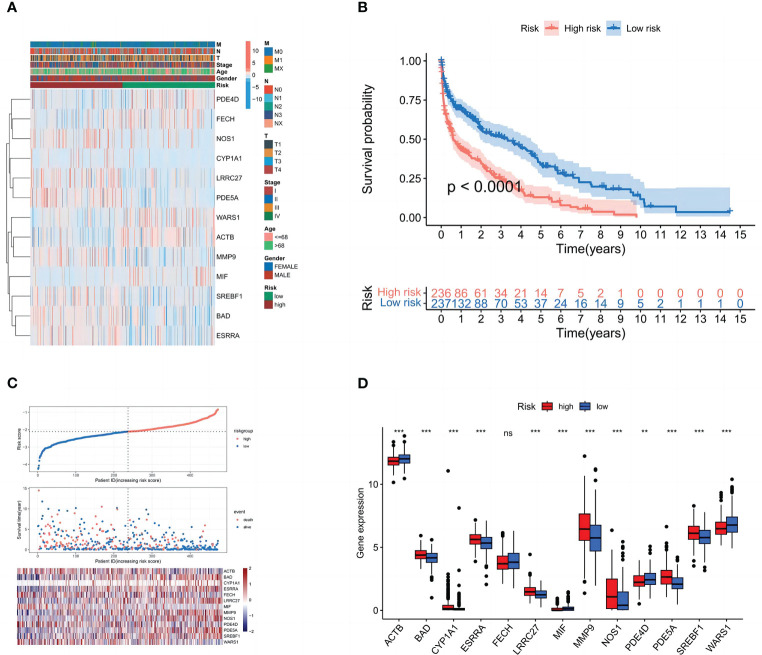
Subgroup correlation heat map and survival analysis of high- and low-risk groups. **(A)** Patient clinicopathological characteristics are distributed in different ways. **(B)** Box plot of model gene differential analysis: comparison of model genes differentially expressed in high- and low-risk groups.**(C)** Patient’s OS according to Kaplan-Meier curves for high- and low-risk groups. **(D)** The variation in immune checkpoint gene expression between various populations. ns, P=>0.05: **, P<0.01; ***, P<0.001.

The patients were separated into high- and low-risk groups in order to better examine the prognostic value of the risk signature. The 13 genes in the constructed model were analyzed differentially for each clinical trait and high and low-risk groups, and heat maps were plotted (**Figure 5A**), demonstrating that (BAD, CYP1A1, ESRRA, LRRC27, MMP9, NOS1, PDE5A, and SREBF1) were highly expressed in the high-risk group. We discovered that patients in the high-risk group had a considerably shorter overall survival than those in the low-risk group in the Kaplan-Meier survival analysis. (**Figure 5B**). The risk curves show the relationship between LUSC patients’ risk scores and survival rates, and we discovered that mortality was higher in high-risk patients than in low-risk individuals. The heatmap showed high- and low-risk levels for 13 genes. For example, 8 genes (BAD, CYP1A1, ESRRA, LRRC27, MMP9, NOS1, PDE5A, and SREBF1) were highly expressed in the high-risk group, which was consistent with the prediction of the model (**Figures 5C, D**).

### 3.4 Independent analysis of prognostic factors

**Figure 6:**
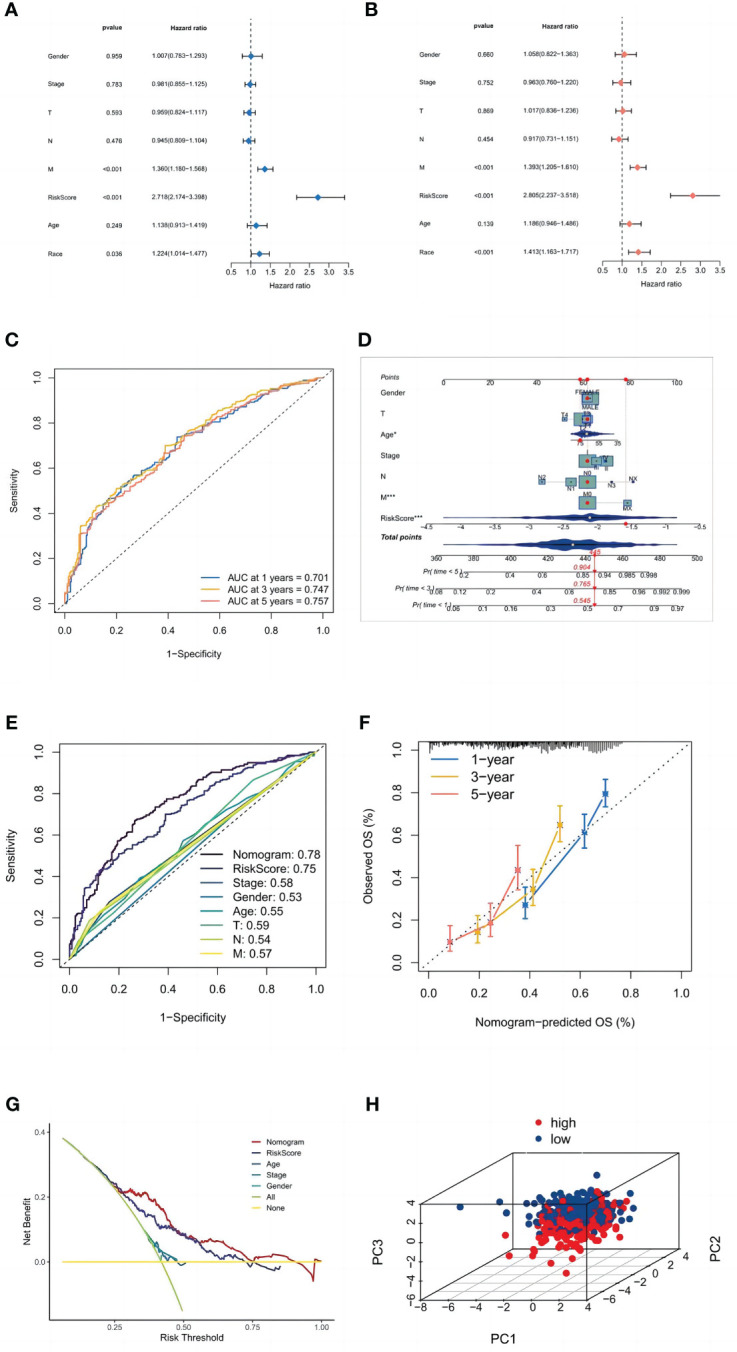
Validation of the model. **(A)** Univariate and **(B)** multivariate Cox regression analyses. **(C)** ROC curves of 1, 3, and 5 years. **(D)** The nomogram prediction model. **(E)** ROC curves of risk scores and clinical characteristics. **(F)** A calibration curve. **(G)** A DCA of the nomogram prediction model and the TNM staging system. **(H)** Principal component analysis of high and low-risk groups.

Univariate and multivariate Cox regression analyses were performed to determine whether the risk signature has the potential to be a prognostic factor independent of other clinical parameters (**Figures 6A, B**). The risk score (HR = 2,805, 2.237–3.518; P<0.001) was significantly linked with OS in multivariate Cox regression, demonstrating that the risk signature is an independent prognostic factor for LUSC patients. Additionally, we evaluated the risk score’s predictive accuracy using ROC curves. The AUC for the risk score was 0.75, which was higher than those for age (0.55), gender (0.53), and stage (0.58). While in the LUSC cohort, the AUCs for 1-, 3-, and 5-year OS were 0.701, 0.747, and 0.757 (**Figure 6C**), indicating that the signature has trustworthy diagnostic applicability.

### 3.5 Construction of nomogram and validation of clinical subgroups and PCA

Age, gender, TNM stage, T stage, M stage, N stage, and the risk score from the signature were used to construct a nomogram (**Figure 6D**) that could accurately predict the 1-, 3-, and 5-year OS of LUSC patients. Furthermore, ROC curves and decision curves were used to test the effectiveness of nomograms (**Figures 6E, F**). The nomogram’s AUC was 0.780, which was higher than the risk score of 0.75 and indicates that the nomogram has reliable diagnostic significance. Then, decision curve analysis(DCA) demonstrated that the nomogram outperformed the other six molecular categorization techniques in terms of clinical net benefit (**Figure 6G**). Finally, we used PCA to look at the distribution of risk genes among patients, and the results showed that these genes could be relied upon to generate the signature (**Figure 6H**).

### 3.6 Gene Ontology and Kyoto Encyclopedia of Genes and Genomes pathway analysis

**Figure 7:**
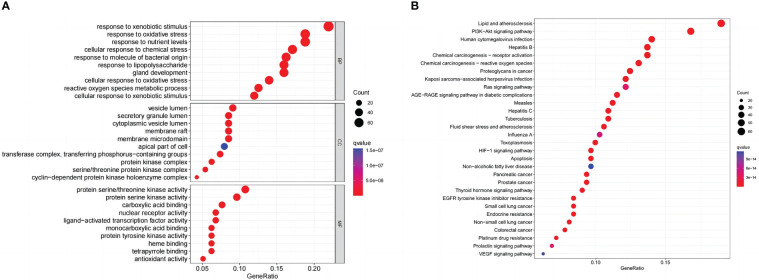
Naringenin functional characterization against COVID-19/LUSC. **(A)** GO analysis of naringenin and COVID-19/LUSC intersecting genes. **(B)** KEGG analysis of naringenin and COVID-19/LUSC intersecting genes.

GO and KEGG enrichment analyses on common targets were performed to investigate the biological activities and pathways of *naringenin* against COVID-19/LUSC. As a consequence, 3133 GO terms were highlighted (BP: 2793, CC: 92, and MF: 248), as well as 183 KEGG pathways. The top 10 GO terms of each ontology and the top 30 KEGG pathways are presented as bubble charts (**Figures 7A, B**). Representative BP terms included the response to xenobiotic stimulus, cellular response to chemical stress, response to oxidative stress, response to nutrient levels, response to lipopolysaccharide, response to molecule of bacterial origin, cellular response to xenobiotic stimulus, reactive oxygen species metabolic process, cellular response to oxidative stress, gland development, etc. Representative CC terms included protein kinase complex, cyclin-dependent protein kinase holoenzyme complex, vesicle lumen, secretory granule lumen, cytoplasmic vesicle lumen, etc. Representative MF terms included nuclear receptor activity, ligand-activated transcription factor activity, carboxylic acid binding, monocarboxylic acid binding, protein serine/threonine kinase activity, protein tyrosine kinase activity, etc. In addition, representative pathways included the PI3K-Akt signaling pathway, the VEGF signaling pathway, the HIF-1 signaling pathway, Pancreatic cancer, human cytomegalovirus infection, toxoplasmosis, prostate cancer, Chemical carcinogenesis—receptor activation, non-small cell lung cancer, chemical carcinogenesis—reactive oxygen species, Kaposi sarcoma-associated herpesvirus infection, proteoglycans in cancer, small cell lung cancer, endocrine resistance, apoptosis; colorectal cancer, thyroid hormone signaling pathway, tuberculosis, etc. In conclusion, Go and KEGG analysis highlighted that *naringenin*’s anti-inflammatory, antiviral, and anticancer properties are important targets/pathways in COVID-19/LUSC treatment.

### 3.7 PPI network analysis

**Figure 8:**
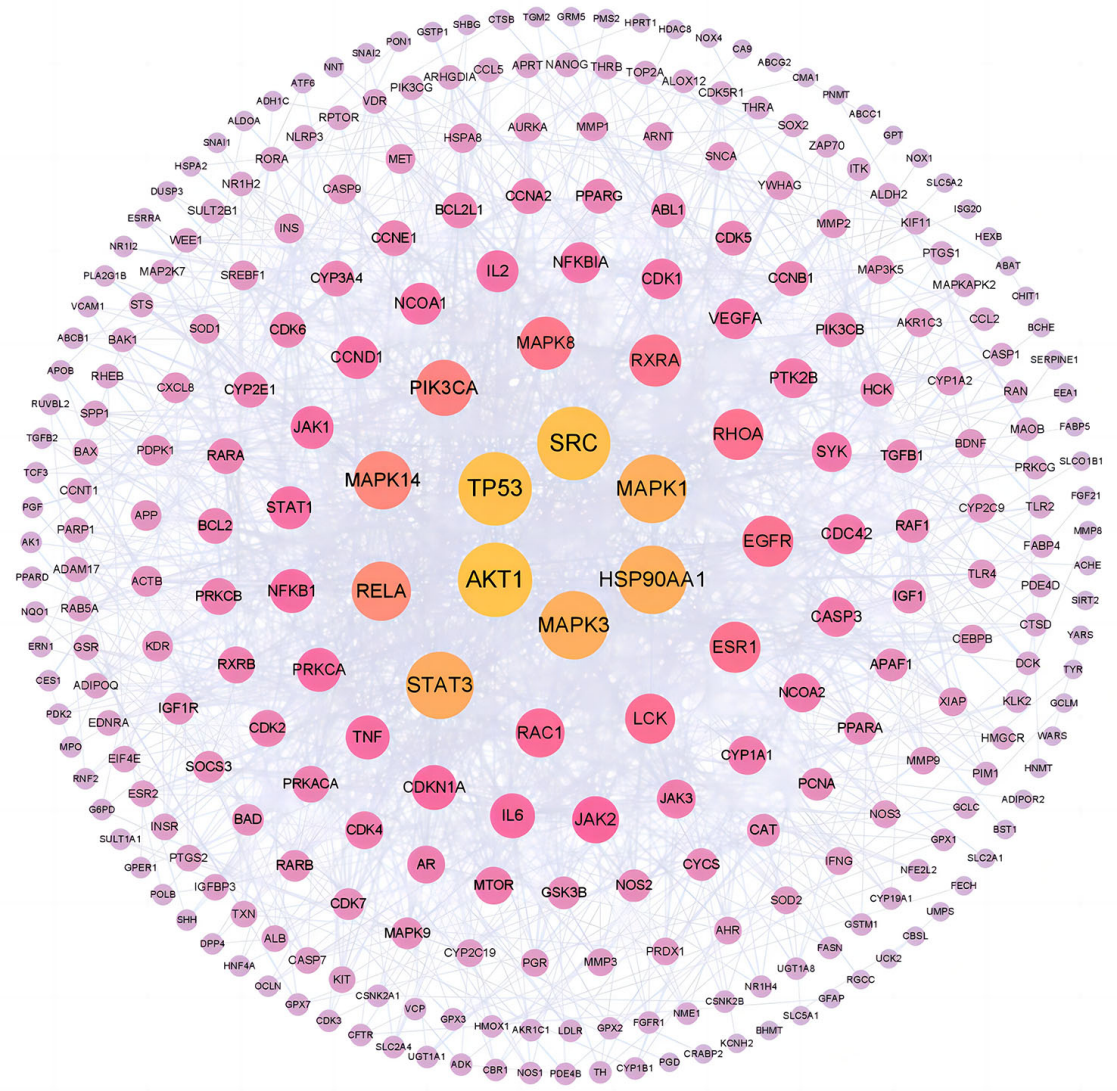
PPI network for hub targets of naringenin against COVID-19/LUSC.

The PPI network of common targets contained 268 nodes and 1,358 edges, which represented targets and interactions between targets, respectively. In the fight against COVID-19/LUSC, a node with a darker color and a larger form is more important. According to **Figure 8**, the top 6 targets with the highest degree values were AKT1 (degree = 55), TP53 (degree = 54), SRC (degree = 54), MAPK1 (degree = 49), MAPK3 (degree = 49), and HSP90AA1 (degree = 49). Consequently, molecular docking with *naringenin* was performed using AKT1, TP53, SRC, MAPK1, MAPK3, and HSP90AA1 as the hub targets for *naringenin* to cure COVID-19/LUSC.

### 3.8 Molecular docking

**Figure 9:**
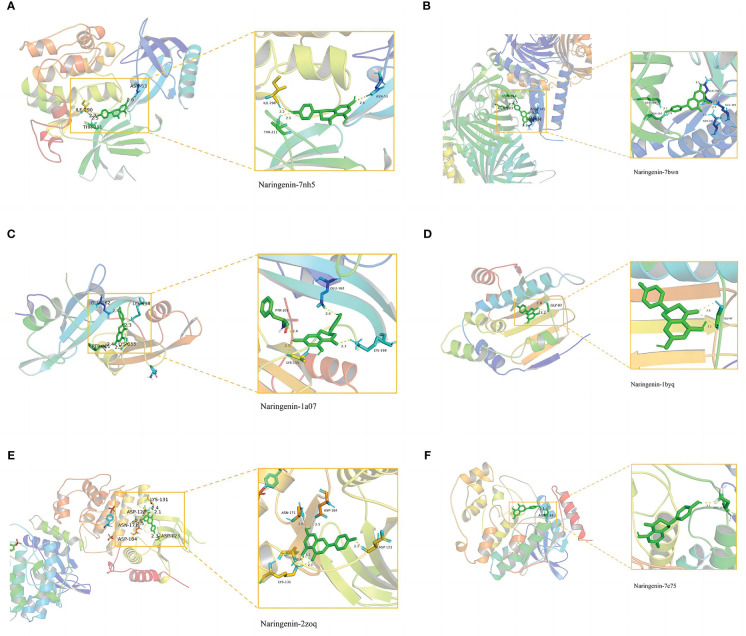
Molecular docking of naringenin and the targets AKT1, TP53, SRC, HSP90AA1, MAPK3, and MAPK1. **(A)** The binding of naringenin with the 7nh5 protein of AKT1. **(B)** The binding of naringenin with the 7bwn protein of TP53. **(C)** The binding of naringenin with the 1a07 protein of SRC. **(D)** The binding of naringenin with the 1byq protein of HSP90AA1. **(E)** The binding of naringenin with the 2zoq protein of MAPK3. **(F)** The binding of naringenin with the 7e75 protein of MAPK1.

In general, stronger binding conformations and higher interaction probabilities result from lower binding energies. Related studies revealed that binding energy< 0 kJ/mol imply spontaneous binding, and -5.0 kJ/mol or lower indicates good binding activity (66). We analyzed the possible binding of *naringenin* with the six COVID-19/LUSC hub targets (AKT1, TP53, SRC, HSP90AA1, MAPK3, and MAPK1) identified previously and found that all of the docking results showed strong binding activity. Amino acid residues ASN-53, ILE-290, and THR-211 in AKT1, amino acid residues GLN-183, ASN-164, GLN-248, GLU-349, and ASN-345 in TP53, amino acid residues GLU-162, PTR-101, LYS-155, and LYS-198 in SRC, amino acid residues GLY-97 in HSP90AA1, amino acid residues LYS-131, ASP-128, ASN-171, ASP-184, and ASP-123 in MAPK3, amino acid residues ASN-144 in MAPK1, and *naringenin* form hydrogen bonds tightly. Overall, our findings demonstrated the high affinity between *naringenin* with AKT1, TP53, SRC, HSP90AA1, MAPK3, and MAPK1 (**Figures 9A–F**).

## 4 Discussion

COVID-19 is a serious, rapidly spreading infectious disease that can be fatal (67). At this time, it is known that older age, male sex, diabetes mellitus, obesity, cardiovascular disease, and cancer are risk factors for COVID-19 infection and serious outcomes (68, 69). Cancer patients are vulnerable to respiratory viruses due to immunosuppression caused by the disease or therapy. This susceptibility has been demonstrated with influenza, which has been linked to a higher mortality rate in people with solid and hematologic cancer (70). Lung cancer patients from COVID-19 had the highest death risk compared to those with other cancers, which is most likely related to advancing age, a decline in lung reserve, concomitant conditions, and cancer treatment (71). We investigated the potential usefulness and molecular mechanisms of *naringenin* for COVID-19/LUSC using bioinformatics and system pharmacology methodologies based on the extensive biological properties of *naringenin*, including anti-inflammatory, anti-tumor, and antiviral. This might offer a fresh option for raising the survival rate of COVID-19/LUSC patients and stopping the spread of SARS-COV-2 through the digestive system.

In the present study, the potential mechanism and prognostic value of *naringenin* in COVID-19/LUSC were comprehensively investigated through network pharmacology and bioinformatic analyses, and we acquired 354 common targets of *naringenin* against COVID-19/LUSC. According to GO and KEGG enrichment analysis, the mechanisms for *naringenin* treatment of COVID-19/LUSC may be related to oxidative stress, immunoregulation, apoptosis, antiviral, anti-inflammatory, anti-cancer, and associated signaling pathways such as PI3K-Akt, HIF-1, and VEGF. Additionally, molecular docking demonstrated that *naringenin* and the top 6 COVID-19/LUSC related-target proteins had strong binding activity. As a result, the findings illustrate that *naringenin* holds a lot of promise as a treatment for COVID-19/LUSC.

### 4.1 Excellent prognostic analysis of LUSC patients with COVID-19

We first collected 13422 COVID-19 targets, 568 *naringenin* targets, and 22232 LUSC targets, and then further screened out 354 common targets, then selected 472 patient samples for follow-up prognostic analysis. Subsequently, a thirteen-gene signature containing ACTB, BAD, CYP1A1, ESRRA, FECH, LRRC27, MIF, MMP9, NOS1, PDE4D, PDE5A, SREBF1,and WARS1 was developed through univariate and multivariate Cox analyses, LUSC patients with COVID-19 may benefit from independent prognostic variables. Additionally, multivariate ROC analysis demonstrated that risk ratings were significantly more accurate than conventional pathological prognostic variables in predicting OS. Analysis of the nomogram revealed that the prognostic signature may be utilized to predict the outcomes of LUSC patients who have the COVID-19 mutation.

### 4.2 Hub targets could be set off by *Naringenin* to fight COVID-19/LUSC

In the PPI network of shared targets, which we first obtained, there were 268 nodes and 1,358 edges for the 354 common targets of *naringenin* and COVID-19/LUSC. In addition, we chose the top six targets in the PPI network as well as essential proteins connected to COVID-19/LUSC for molecular docking with *naringenin*. There were significant variations in the expression of the top six target genes, which included AKT1, TP53, SRC, MAPK1, MAPK3, and HSP90AA1. A member of the serine/threonine protein kinase subfamily known as Akt1 (72), viral protein synthesis is facilitated by overexpressed AKT1, and silencing of AKT1 results in reduced viral RNA expression, suppression of viral capsid protein synthesis, and virus release (73, 74). According to a study, which supports our hypothesis, AKT1 inhibition lowers viral yields in Huh7 cells infected with SARS-CoV-2 (75). An important aspect of the development of cancer is cell migration and motility, in different malignancies, Akt1 activation and expression leads to the advancement of carcinogenesis and metastasis (76). By controlling the expression of certain genes, such as those in the Akt signaling pathway, Akt1 plays a significant role in the development of tumors. Furthermore, AKT1 regulates innate immunity, which affects macrophage immunological function and the activated phenotype. AKT1 activation increases inflammatory and metabolic responses, making it a suitable target for COVID-19 therapy (57, 77, 78). The p53 protein, expressed by the TP53 gene, has been dubbed the “guardian of the genome” due to its involvement in responding to DNA damage by inducing cell cycle arrest, apoptosis, and/or senescence (79). The potential for TP53 gene therapy through SGT-53 to suppress viral infections against the many SARS-CoV-2 variants that have evolved or may develop throughout the COVID-19 pandemic (80). The gene that suppresses tumors One of the most frequently altered genes in human lung cancer is TP53 (81). In addition to causing tumor development, defects in TP53 function impair the response of malignant cells to anticancer drugs, especially those that induce DNA damage (82) and TP53 mutations are more common than average in LUSC (83). Therefore, it is anticipated that TP53 would be crucial in the fight against LUSC (84). When MAPK family members and the MAPK-STAT3 axis are activated, inflammatory factors such as IL-1, TNF-, and IL-6 are overexpressed (85). The MAPK1 signaling pathway has been associated to ALI/ARDS inflammation (86). Many cytokines, including IL-1, TNF-, and IL-6, play important roles in ALI/ARDS, primarily *via* the MPAK1 signal transduction pathway (87), The study discovered that an inhibitor of MAPK3/MAPK1 following carrageenan induced a reduction in all inflammation parameters assessed, which could be effective in the treatment of numerous inflammatory illnesses (88),Therefore, we have reason to believe that regulating MAPK pathway has positive significance for improving the expression of inflammatory factors in COVID-19. Meanwhile, MAPK1 restoration inhibited the proliferation, migration, and invasion of NSCLC cells (89). HSP90AA1 is a gene that promotes squamous cell lung cancer progression (90) and has an important regulatory role in non-small cell lung cancer (91). Simultaneously, the study discovered that decreasing HSP90AA1 expression can lower inflammatory factors, ROS generation, cell apoptosis rate, and autophagy-related proteins (92). These results further suggested that *naringenin* could be an effective pharmaceutical target for these intersecting genes against LUSC and COVID-19.

### 4.3 The critical mechanisms for *naringenin* to combat COVID-19/LUSC

The mechanism of *naringenin* against COVID-19/LUSC is strongly linked to oxidative stress, immunoregulation, apoptosis, antiviral, anti-inflammatory, anti-cancer, and related pathways including PI3K-Akt, HIF-1, and VEGF signaling pathway, according to the findings of GO and KEGG enrichment study. Excessive oxidative stress impairs immune system performance, increasing the risk of SARS-CoV-2 viral invasion in the body (93). Cancer, neurodegeneration, cardiovascular conditions, diabetes, and other illnesses also can be brought on by abnormal oxidative stress (94). The major characteristic of cancer cells is reduced apoptosis, which is accomplished by modifying important signaling molecules or pathways. The proto-oncogene Akt, whose expression and activation are increased in a variety of cancers, including LUSC, contributes to the resistance of cancer cells to chemotherapy and radiation therapy (95, 96). High basal levels of PI3K-Akt activation in clinical samples suggested an aggressive type of LUSC (97). The PI3K-Akt signaling pathway is specifically activated by CD147, which is crucial in the entry of SARS-CoV-2 into cells, and its shutting down will prevent some viruses from entering cells (98). Furthermore, by inhibiting the PI3K/AKT/mTOR signaling pathway, SARS-CoV-2 spike pseudovirions promote the appearance of autophagy, which in turn initiates apoptosis (99). Another hallmark of COVID-19 is tissue hypoxia, which is related to overexpression of the HIF-1along with their immunometabolic and immune-response implications (100). HIF-1, as one of the hypoxia signal transcription factors, regulates the expression of genes involved in metabolism in macrophages and T cells, promoting an inflammatory response (101, 102). The HIF-1 pathway regulates oxidative stress, hypoxia, and inflammation, and its activity may promote SARS-CoV-2 infection and affect a variety of physiological processes (103). HIF-1 is a fibroblast master regulator of lipid metabolism that contributes to a tumor-promoting phenotype in lung fibroblasts (104). Therapy for lung cancer may benefit from focusing on the HIF-1/SCD1 axis in CAFs (65). HIF-1 is thus a potential target in research against COVID-19/LUSC due to the importance of HIF-1 stabilization in tumor progression. As is generally known, VEGF is the best-characterized mediator of angiogenesis at the molecular level (105). Cancer cells that overexpress VEGF exhibit enhanced tumorigenicity, invasiveness, proliferation, and EMT features (106). Therefore, VEGF coordinates non-angiogenic events that are crucial for the early spread of tumors (107). When VEGF levels are elevated, it results in high permeability, edema, and tissue injury, which are the pathophysiologic causes of acute lung injury in COVID-19 patients (108, 109). The VEGF signaling pathway, on the other hand, raises angiotensin II(Ang II) levels to promote inflammation, while Ang II can also raise VEGF to promote the release of inflammatory cytokines (110). In conclusion, *Naringenin* may have an antiviral, anti-inflammatory, and anti-cancer effect by regulating oxidative stress, apoptosis, HIF-1, PI3K-Akt, and VEGF signaling pathways in order to alleviate the clinical symptoms of COVID-19/LUSC patients.

Last but not least, molecular docking data showed that *naringenin* has strong binding capabilities with the six COVID-19/LUSC targets, demonstrating that *naringenin* can effectively bind to specific proteins connected to COVID-19/LUSC. According to network pharmacology, *naringenin* can be used to treat SARS-CoV-2-infected patients with LUSC. More experimental investigation, however, is required to confirm and investigate the expected targets and their regulatory processes.

To sum up, We created a trustworthy predictive model for patients with LUSC and COVID-19 as well as many possible treatment targets of COVID-19/LUSC. Further, through the pharmacological actions and possible targets that have been identified, *naringenin* may be used to treat COVID-19/LUSC, including immunomodulation, antivirals, anti-inflammation, etc. Additionally, we were able to find direct binding sites that had a strong affinity for *naringenin* against COVID-19/LUSC, which gave the justification for further clinical trials as well as the proof for its use in clinical settings.

### 4.4 Strengths and limitations

Notably, our study offered some novel insights into *naringenin* in the therapy of COVID-19/LUSC and suggested plausible molecular pathways and prospective pharmacological targets of *naringenin* for the first time. However, a few remaining issues with our study’s limitations must be resolved. Since the results of this study were not verified in actual LUSC patients with COVID-19, future confirmation of these findings will need the recruitment of actual LUSC patients with COVID-19. Second, additional *in vivo* and *in vitro* studies are necessary to confirm the hypothesized mechanisms and pharmacological targets in order to confirm the potential therapeutic application of *naringenin* for COVID-19/LUSC.

## Data availability statement

The datasets presented in this study can be found in online repositories. The names of the repository/repositories and accession number(s) can be found in the article/**Supplementary Material**.

## Author contributions

S-fZ, KW, and X-fH conceived and designed this research. W-yW and XJ wrote the manuscript and participated in the design of the study. W-yW and XJ were responsible for the bioinformatics analysis and network construction. P-qX and W-xS carried out the data analysis and data interpretation. PW helped to modify the manuscript. All authors contributed to the article and approved the submitted version.
